# Does Nomegestrol Acetate Plus 17β-Estradiol Oral Contraceptive Improve Endometriosis-Associated Chronic Pelvic Pain in Women?

**DOI:** 10.1089/jwh.2020.8291

**Published:** 2020-09-14

**Authors:** Salvatore Caruso, Antonio Cianci, Marco Iraci, Valentina Fava, Salvatore Di Pasqua, Stefano Cianci

**Affiliations:** ^1^ Department of General Surgery and Medical Surgical Specialties, University of Catania, Catania, Italy.; ^2^ Research Group for Sexology, University of Catania, Catania, Italy.; ^3^ Department of Woman, Child and General and Specialized Surgery, University of Campania “Luigi Vanvitelli,” Naples, Italy.

**Keywords:** combined oral contraceptives, endometriosis-associated chronic pelvic pain, nomegestrol acetate, quality of life, sexual activity, 17β-estradiol

## Abstract

***Background:*** To evaluate the effects of a 24/4 regimen combined oral contraceptive (COC) containing 1.5 mg 17β-estradiol (E2) and 2.5 mg nomegestrol acetate (NOMAC) compared to on-demand nonsteroidal anti-inflammatory drugs (NSAIDs) on women affected by endometriosis-associated chronic pelvic pain (the primary end point) and their quality of life (QoL) and sexual function (the secondary end points).

***Materials and Methods:*** Ninety-nine women on E2/NOMAC constituted the study group; and 63 women on NSAIDs constituted the control group. The visual analogic scale was used to measure the levels of pelvic pain, dysmenorrhea, and dyspareunia. To assess their QoL, sexual function, and sexual distress, the Short Form-36 (SF-36), the Female Sexual Function Index (FSFI), and the Female Sexual Distress Scale (FSDS) were used, respectively. The study included two follow-ups at 3 and 6 months.

***Results:*** Improvement in chronic pelvic pain was observed in the study group at both the 3- and 6-month follow-ups (*p* < 0.001). SF-36, FSFI, and FSDS had a similar trend at the 3- and 6-month follow-ups (*p* < 0.001). Women on NSAIDs did not report any reduction in pain symptoms or improvement in QoL (*p* ≤ 0.4). However, they had a limited improvement of their FSFI and FSDS (*p* < 0.001). The improvement of the pain symptoms, QoL, FSFI, and FSDS, was more evident in women on E2/NOMAC than in those on NSAIDs, when the study group and control group values were compared at the 3- and 6-month follow-ups (*p* < 0.001).

***Conclusions:*** Women on E2/NOMAC COC showed a better reduction of endometriosis-associated chronic pelvic pain and an improvement of their QoL and sexual activity than those of the women on NSAIDs.

## Introduction

Approximately 10% of women of reproductive age are affected by endometriosis-associated chronic pelvic pain.^1^ Women frequently experience dysmenorrhea, dyspareunia, dyschezia, fatigue, and infertility together with chronic pelvic pain.^2–4^

Progestins taken orally^5–7^ or by subdermal implant,^8^ intrauterine device^9,10^ administration, or estrogen–progestin combined hormonal contraceptives (CHCs)^3^ are normally used to counteract, but not to cure, chronic pelvic pain symptoms due to the effects of ovarian estrogenic secretion on the endometrium-like tissue developing outside the uterus. In fact, when women discontinue their usage, pelvic pain usually reappears.^11^

It is customary for women affected by chronic pelvic pain to take on-demand nonsteroidal anti-inflammatory drugs (NSAIDs).^12^ Women commonly adopt hormonal treatment when their doctor informs them that they are infertile and/or affected by endometriosis. However, the first step in prescribing a drug for chronic pelvic pain is to understand the needs of each subject. In fact, women without the desire for procreation are more likely to use CHCs.

Outcomes depend on the specific dose, formulation, delivery system, or regimen.^13,14^ Conventional 21/7 regimens can fail during the 7-day hormone-free interval producing limited benefits on pain symptoms; therefore, continuous or extended CHC regimens were introduced.^15^ In fact, women on conventional CHCs may still experience headaches, pelvic pain, and mood changes during the hormone-free interval, mainly due to the raised estradiol levels and increased number of follicles induced by the increasing follicle-stimulating hormone levels during the hormone-free interval.^16^

Recently, a new oral CHC containing 1.5 mg 17β-estradiol (E2) with 2.5 mg nomegestrol acetate (NOMAC) has been used with 24 days of active pill use followed by a 4-day hormone-free interval regimen.^17^ NOMAC has a long half-life (45–50 hours) compared with other contraceptive progestins, and thus, it is able to cover the 4-day hormone-free interval by its steroidal effects; consequently, its contraceptive efficacy could be more compared with progestins with a shorter half-life.^18^

This study aimed to evaluate the effects of an E2/NOMAC contraceptive pill on the pain symptoms of women affected by endometriosis-associated chronic pelvic pain without the desire for procreation (the primary end point). Moreover, the quality of life (QoL) and sexual function were investigated (the secondary end points).

## Materials and Methods

The departmental institutional review board approved the study. The study protocol conformed to the ethical guidelines of the 1975 Helsinki Declaration. Informed written consent was obtained from each woman before entering the study, and they did not receive payment of any kind. The time of enrollment was from October 2017 to March 2019.

Two hundred fifty-five women aged 18–39 years old (mean age 26.7 ± 7.6), affected by chronic pelvic pain, were invited to participate in a comparative, open-label prospective study at the Chronic Pelvic Pain Clinic of the Gynecological Unity, Department of General Surgery and Medical Surgical Specialties, University of Catania, Italy. Each woman had been using on-demand NSAIDs from 15 months to 8 years. Before enrollment, medical, surgical, and medication history were assessed to ensure study eligibility based on inclusion and exclusion criteria for combined oral contraceptive (COC) usage. Moreover, women under gonadotropin releasing hormone analog treatment within the last 6 months or hormonal contraceptive treatment within the last 3 months were excluded.

Furthermore, women had to be sexually active, having had at least one sexual activity during the month before counseling, without desiring procreation in the following 6 months after the start of the study. Women with a history of nonorganic sexual dysfunction or having a partner affected by sexual dysfunction were excluded. A psychosexual investigation was performed by S.C.

Moreover, physical and gynecological examinations and transvaginal sonography (TVS), to rule out rectovaginal endometriosis ovarian endometria or adenomyosis, were performed. Based on the European Society of Human Reproduction and Embryology guidelines,^2^ only women affected by chronic pelvic pain with a clinical suspicion of endometriosis, such as dysmenorrhea, noncyclical chronic pelvic pain, or deep dyspareunia, were included. Consequently, 93 (36.4%) women with ultrasound-based evidence of endometriosis were excluded from participating in the study. Of these 33 (12.9%), 31 (12.2%) and 29 (11.3%) women were diagnosed by TVS to have rectovaginal endometriosis, ovarian endometrioma, or adenomyosis, respectively. Finally, 162 (63.6%) women participated in the study.

After having received counseling on the benefits of E2/NOMAC COC usage in an extended 24/4 regimen, 99 (61.1%) women accepted to take the pill, constituting the study group. The remaining 63 (38.9%) women refused to use the pill or any other hormonal treatment, preferring to continue their previous NSAID on-demand therapy. They accepted to constitute the control group after having signed a new informed consent form. All the women provided the names of the NSAIDs they were usually taking to relieve their pelvic pain: ibuprofen, paracetamol, diclofenac, ketorolac, and ketoprofen, were taken orally; participants were allowed to take only NSAIDs contained in this list. No opioids or other types of NSAIDs could be used or prescribed.

### Instruments

The visual analogic scale (VAS) was used to define endometriosis-associated pain,^19^ nominally chronic pelvic pain, dysmenorrhea, and dyspareunia.

The Short Form-36 (SF-36) validated questionnaire was used to assess QoL.^20^ The questionnaire contains 36 questions grouping four categories of somatic aspects [physical activity (10 items), physical role (4 items), bodily pain (2 items), general health (6 items)] and four mental aspects [vitality (4 items), social activity (2 items), emotional role (3 items), and mental health (5 items)]. Women were instructed to place a mark on a 0–100 scale for each item that best corresponded to their feelings, from the lowest to the highest score of a given category of the QoL questionnaire. Thereafter, the sum of all items of each category was made. Mean values were calculated on the basis of individual items within a given category. Consequently, somatic and mental scale scores were obtained, with higher scores indicating better functioning.

Each woman underwent a sexual history interview. To define sexual dysfunction, the definition and classification of the second report of the international consensus development conference were used.^21^

Sexual behavior was assessed using the self-administered Female Sexual Function Index (FSFI) validated in the Italian gynecological population.^22^ The FSFI consists of six domains, which include desire (two items), arousal (four items), lubrication (four items), orgasm (three items), satisfaction (three items), and pain (three items), answered on a 5-point Likert scale, ranging from 0 (no sexual activity) or 1 (never/very low) to 5 (always/very high). A score is calculated for each of the six domains, and the total score is obtained summing all the items. The total score range is 2–36. An FSFI cutoff of ≤26.55 is usually accepted for diagnosis of sexual dysfunction in women within a wide age range.

Moreover, for diagnosis of sexual dysfunction an essential element is the requirement that the condition causes significant personal distress for the woman. Therefore, the Female Sexual Distress Scale (FSDS) was used.^23^ The FSDS consists of 12 items, with a maximum score of 48. An FSDS score of ≥15 corresponds to clinically significant distress. We considered women with an FSFI score of ≤26.55 to be affected by sexual dysfunction if they also had an FSDS score of 15 or greater.

The study included two follow-ups at 3 and 6 months. All questionnaires were administered to both groups at the baseline evaluation and at each follow-up. Moreover, each woman received a diary to record daily sexual events, covering behaviors from self-stimulation to arousal with their partner and actual intercourse, indicated as “yes” or “no”; adverse events, namely spotting, nausea, or breast tenderness; and the characteristics of withdrawal and intracyclic bleeding. The recall period to check the events recorded above was at the end of each COC blister (about every 30 days).

At the end of the study, the women of the study group and those of the control group were asked to rate their satisfaction using E2/NOMAC COC and NSAIDs, respectively, indicating the following: very satisfied, somewhat satisfied, neither satisfied nor dissatisfied, dissatisfied, or very dissatisfied. Each woman on E2/NOMAC COC was informed that the pill and its regimen could cause amenorrhea.

### Statistical analysis

Analysis of variance was used to compare the demographic and clinical data and VAS, SF-36, FSFI, and FSDS scores between the two groups. Paired Student's *t*-test was used to compare the values obtained at baseline with those of the follow-ups from the VAS, SF-36, FSFI, and FSDS scores. The values are presented as mean ± standard deviation. The result was statistically significant when *p* < 0.05. Statistical analysis was carried out using the Primer of Biostatistics statistical computer package (Glantz SA, New York, USA: McGraw-Hill, Inc., 1997).

## Results

Table 1 shows the demographic characteristics of the study group and the control group at baseline. At the 3-month follow-up, 10 (10.1%) women of study group discontinued COC due to spotting; at the 6-month follow-up the dropout rate was 12.1% (12 women). Therefore, 77 (77.8%) women completed the study. However, in their daily diary women recorded mild adverse events at the 3-month follow-up, which did not provoke discontinuation, namely spotting (16 = 16.2%), nausea (12 = 12.2%), or breast tenderness (13 = 13.2%). Finally, 17 (17.2%) and 29 (29.3%) women had amenorrhea at the 3-month follow-up and the 6-month follow-up, respectively.

**Table 1.** Demographic Characteristics

	Treated group (*n* = 99),* n *(%)	Control group (*n* = 63),* n *(%)	*p*
Age range (years)	18–38	18–35	1
Mean age	26.4 ± 6.8	28.2 ± 6.2	0.22
BMI kg/m^2^	21.8 ± 4.7	23.4 ± 6.1	0.17
Age at menarche	12.6 ± 2.4	12.8 ± 2.8	0.72
Menstrual cycle length (days)	26 to 32	25 to 30	1
Duration of menses (days)	4.5 ± 2.2	5.4 ± 1.8	0.06
Chronic pelvic pain	99 (100)	63 (100)	1
Dysmenorrhea	73 (73.7)	47 (74.6)	1
Dyspareunia	62 (62.6)	40 (63.5)	1
Parity			
Nulliparous	74 (74.7)	46 (73.1)	1
One or more children	25 (25.3)	17 (26.9)	1
Cigarette smoking			
Nonsmoker	84 (84.8)	53 (84.1)	1
Current smoker	15 (15.2)	10 (15.9)	1
Daily cigarettes	15.3 ± 3.2	13.7 ± 4.2	0.05
Current coffee drinker	93 (93.9)	53 (93.6)	1
Daily cups of coffee	2.5 ± 2.9	2.1 ± 2.8	0.53
Systolic blood pressure (mmHg)	117.5 ± 9.5	119.2 ± 5.7	0.35
Diastolic blood pressure (mmHg)	71.3 ± 7.8	69.5 ± 7.5	0.29
Heart rate ( × minutes)	72.5 ± 8.6	68.6 ± 5.5	0.01

BMI, body mass index.

The dropout rate of the control group was 9.5% (6 women) and 28.6% (18 women) at the 3- and 6-month follow-ups, respectively. Consequently, 39 (61.9%) women completed the study. They did not record any adverse events during the usage of on-demand NSAIDs, either at the 3- or 6-month follow-ups.

**FIG. 1.**
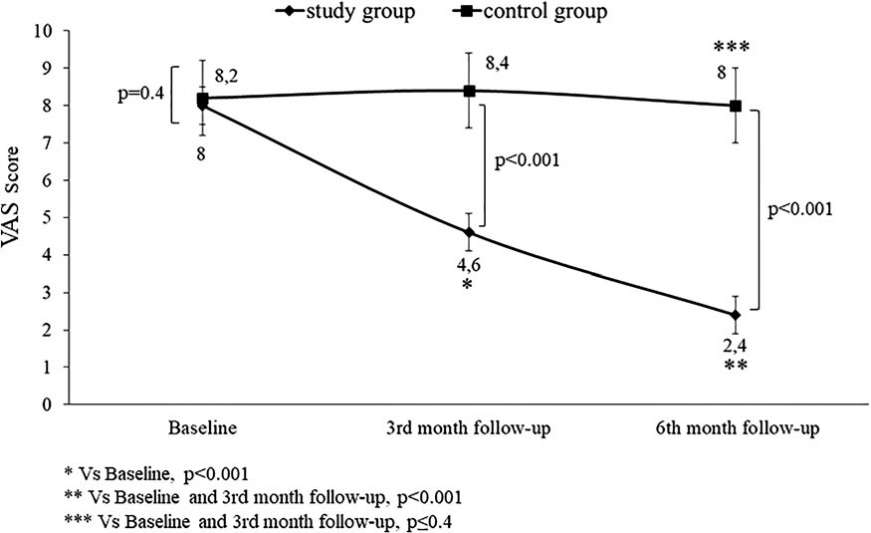
Chronic pelvic pain, dysmenorrhea, and dyspareunia of women with endometriosis-associated pain symptoms at baseline and at 3 and 6 months of 24/4 regimen 17β-estradiol (1.5 mg) and NOMAC (2.5 mg) combined oral contraceptive (study group) and of women using on-demand NSAIDs (control group). NOMAC, nomegestrol acetate; NSAIDs, nonsteroidal anti-inflammatory drugs; VAS, visual analogic scale.

The intragroup analysis showed an improvement of the VAS score at the 3- and 6-month follow-ups of women on E2/NOMAC (*p* < 0.001). No change was observed in the control group from baseline to both the follow-ups (*p* ≤ 0.4). The intergroup comparison between baseline values showed no differences (*p* ≤ 0.4). On the contrary, the comparison was statistically significant at the 3-month (*p* < 0.001) and the 6-month follow-ups (*p* < 0.001) (Fig. 1).

**FIG. 2.**
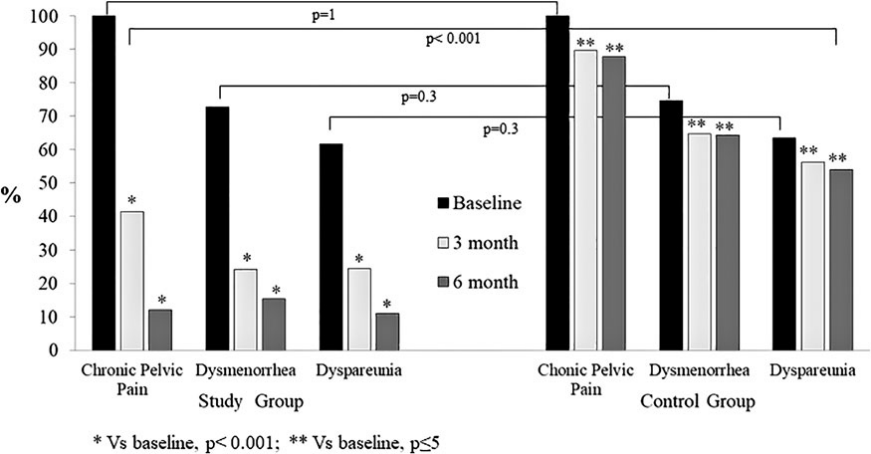
Chronic pelvic pain, dysmenorrhea, and dyspareunia of women with endometriosis-associated pain symptoms at baseline and at 3 and 6 months of 24/4 regimen 17β-estradiol (1.5 mg) and NOMAC (2.5 mg) combined oral contraceptive (study group) and of women using on-demand NSAIDs (control group).

Women on E2/NOMAC had a reduction of chronic pelvic pain of 58.7 and 87.7%, dysmenorrhea of 46.4 and 57.9%, and dyspareunia of 21.8 and 43.7% at the 3- and the 6-month follow-ups compared to baseline, respectively (*p* < 0.001). On the contrary, the women of the control group had a reduction of chronic pelvic pain (not statistically significant) of 10.3% and 12.1%, dysmenorrhea of 9.8% and 10.4%, and dyspareunia of 7.9% and 9.5%, at the 3- and 6-month follow-ups compared to baseline, respectively (*p* ≤ 0.5). Finally, no difference was observed between the study group and the control group at baseline (*p* ≤ 0.3). On the contrary, the intergroup differences were statistically significant at the 3- and 6-month follow-ups (*p* < 0.001) (Fig. 2).

**FIG. 3.**
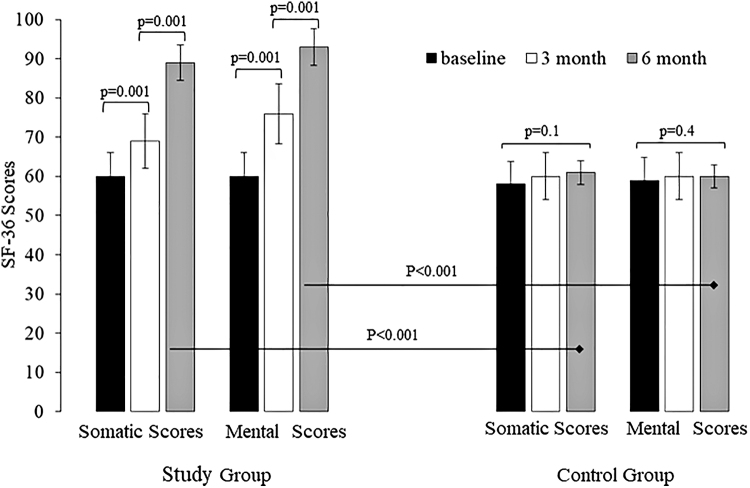
Quality of life of women with endometriosis-associated pain symptoms at baseline and at 3 and 6 months of 24/4 regimen 17β-estradiol (1.5 mg) and NOMAC (2.5 mg) combined oral contraceptive (study group) and of women using on-demand NSAIDs (control group). SF-36, Short Form-36.

The QoL of the women of the study group improved at the 3- and 6-month follow-ups, compared to the baseline scores (*p* < 0.001). On the contrary, the women of the control group did not have any QoL changes during the study period (*p* ≤ 0.4). The intergroup differences were statistically significant at the 3- and 6-month follow-ups (*p* < 0.001) (Fig. 3).

The FSFI and FSDS improved at the 3 (*p* = 0.001) and 6 (*p* = 0.001) month follow-ups in women on E2/NOMAC (Table 2). Even if the women of the control group had improved FSFI scores at both the follow-ups (*p* = 0.001) and the FSDS score at the 6-month follow-up (*p* = 0.001), however, the scores remained under the cutoffs (Table 3). In fact, the intergroup statistical comparison for FSFI and FSDS scores showed statistically significant differences at the 3- and 6-month follow-ups (*p* < 0.001) (Tables 4 and 5).

**Table 2.** Female Sexual Function Index and Female Sexual Distress Scale Scores of Women with Endometriosis-Associated Pain Symptoms at Baseline and at 3 and 6 Months of 24/4 Regimen 17β-Estradiol (1.5 mg) and Nomegestrol Acetate (2.5 mg) Combined Oral Contraceptive

	Baseline (*n* = 99)	3rd month follow-up (*n* = 89)	6th month follow-up (*n* = 77)	p-value, 3rd month vs. baseline	p-value, 6th month vs. baseline
FSFI score	18.3 ± 1.5	27.5 ± 1.6	30.2 ± 1.8	0.001	0.001
FSDS score	18.5 ± 1.5	13.6 ± 1.7	10.2 ± 1.8	0.001	0.001

Values are mean ± SD.

FSDS, Female Sexual Distress Scale; FSFI, Female Sexual Function Index; SD, standard deviation.

**Table 3.** Female Sexual Function Index and Female Sexual Distress Scale Scores of Women with Endometriosis-Associated Pain Symptoms at Baseline and at 3 and 6 Months of Women Using On-Demand Nonsteroidal Anti-Inflammatory Drugs

	Baseline (*n* = 63)	3rd month follow-up (*n* = 57)	6th month follow-up (*n* = 39)	p-value, 3rd month vs. baseline	p-value, 6th month vs. baseline
FSFI score	18.7 ± 1.7	20.8 ± 1.4	22.9 ± 1.8	0.001	0.001
FSDS score	18.3 ± 1.4	17.9 ± 1.7	16.6 ± 1.3	0.1	0.001

Values are mean ± SD.

**Table 4.** Female Sexual Function Index Intergroup Score Comparison of Women with Endometriosis-Associated Pain Symptoms at Baseline and at 3 and 6 Months of 24/4 Regimen 17β-Estradiol (1.5 mg) and Nomegestrol Acetate (2.5 mg) Combined Oral Contraceptive (Study Group) and of Women Using On-Demand Nonsteroidal Anti-Inflammatory Drugs (Control Group)

FSFI score	Baseline	3rd month follow-up	6th month follow-up
Study group	18.3 ± 1.5	27.5 ± 1.6	30.2 ± 1.8
	*n* = 99	*n* = 89	*n* = 77
Control group	18.7 ± 1.7	20.8 ± 1.4	22.9 ± 1.8
	*n* = 63	*n* = 57	*n* = 39
*p*	0.11	0.001	0.001
	95% CI: −0.90 to 0.11	95% CI: 6.18 to 7.21	95% CI: 6.59 to 8.1

Values are mean ± SD.

CI, confidence interval.

**Table 5.** Female Sexual Distress Scale Intergroup Score Comparison of Women with Endometriosis-Associated Pain Symptoms at Baseline and at 3 and 6 Months of 24/4 Regimen 17β-Estradiol (1.5 mg) and Nomegestrol Acetate (2.5 mg) Combined Oral Contraceptive (Study Group) and of Women Using On-Demand Nonsteroidal Anti-Inflammatory Drugs (Control Group)

FSDS score	Baseline	3rd month follow-up	6th month follow-up
Study group	18.5 ± 1.5	13.6 ± 1.7	10.2 ± 1.8
	*n* = 99	*n* = 89	*n* = 77
Control group	18.3 ± 1.4	17.9 ± 1.7	16.6 ± 1.3
	*n* = 63	*n* = 57	*n* = 39
*p*	0.39	0.001	0.001
	95% CI: −0.26 to 0.66	95% CI: −4.87 to −3.73	95% CI: −7.04 to −5.75

Values are mean ± SD.

From the daily diary the monthly frequency of sexual activity improved in women on E2/NOMAC, from 2.1 at baseline to 4.4 at the end of study (*p* < 0.001). On the contrary, the control group did not have any change; in fact, the frequency of sexual activity was 2.2 at baseline and 2.4 at the end of study (*p* = 1).

Finally, 68 (88.3%), 5 (6.5%), and 4 (5.2%) women on E2/NOMAC rated their satisfaction as very satisfied, somewhat satisfied, and neither satisfied nor dissatisfied, respectively. In contrast, 27 (69.2%), 8 (20.5%), and 4 (10.3%) women on NSAIDs rated their satisfaction as neither satisfied nor dissatisfied, dissatisfied, or very dissatisfied, respectively.

## Discussion

The primary end point of this study was to evaluate the efficacy of 6 months' treatment with E2/NOMAC COC in women affected by endometriosis-associated chronic pelvic pain. Women had an improvement in their chronic pelvic pain, dysmenorrhea, and dyspareunia, recorded by VAS at the 3-month follow-up compared to the baseline values; this benefit was maintained until the end of the study. On the contrary, women on NSAIDs did not have benefits during their usage. The progressive reduction of the pain syndrome reported by women on E2/NOMAC COC could have contributed to improve their QoL and their sexual life further (secondary end point). Assessment of their QoL is a crucial parameter to take into account before concluding on the efficacy of a treatment. Improvement in somatic and mental scores by the SF-36 was observed in women on COC at both follow-ups.

Moreover, a gradual improvement of sexual function and a reduction of sexual distress starting from the 3-month follow-up until the 6-month follow-up was observed. The benefits obtained by women on E2/NOMAC were not observed in those using on-demand NSAIDs. Particularly, even if the women on NSAIDs had improved FSFI scores at both the follow-ups and improved FSDS score at the 6-month follow-up, however the scores remained under the cutoffs. Beyond these considerations of intragroup comparison between follow-ups and baseline values, the improvement of the pain symptoms, the QoL, sexual function, and sexual distress was more evident in women on E2/NOMAC than in those on NSAIDs, when the study group and control group values were compared at the 3- and 6-month follow-ups. Moreover, at the end of the study the rate of satisfaction was better (or higher?) in women on E2/NOMAC COC than in those on NSAIDs.

Today, progestogens are used for off-label treatment of endometriosis-associated chronic pelvic pain,^24^ excluding Dienogest (DNG) that has been approved for clinical treatment of endometriosis.^25–27^ Because of its elimination half-life of around 10 hours, DNG is used in continuous regimens.^28^ It is interesting to note that DNG 2 mg daily provides ovulation inhibition, but ovarian activity is not completely suppressed; thus it has not been approved as a contraceptive.^29^

Consequently, women affected by endometriosis-associated chronic pelvic pain that do not desire pregnancy could choose to use an estrogen–progestogen contraceptive, in a continuous regimen. This regimen could be adopted based on the half-life of progestogen and to avoid the hormone-free interval during which the symptoms may reappear. In fact, women on 21/7 conventional COCs may still have pelvic pain during the 7-day hormone-free interval, mainly due to the raised estradiol levels and increasing number of follicles induced by the increasing follicle-stimulating hormone levels during the hormone-free interval.^30^ On the contrary, the effectiveness of combined estradiol valerate with DNG in a 26/2 four-phase formulation pill has been observed.^31^

Currently, DNG could also be used with 30 μg ethinyl estradiol (EE) in a 21/7 combined regimen pill, but this regimen can fail in controlling the chronic pelvic pain; therefore, continuous regimen was introduced,^30^ even if the quantity of EE could act negatively on ectopic endometrium tissue with an excessive proliferative effect and insufficient inhibiting activity of progestogen. In fact, recent investigations on estrogen and progesterone signaling in the healthy endometrium show a complementary activity of the two steroids, the first inducing epithelial proliferation and the second inhibiting estrogen proliferation. This harmonic correlation between the two steroids undergoes dysregulation in endometriosis.^32^

Endometrium-like tissue located outside the uterine cavity is under a continuous inflammatory condition due to progesterone resistance and estrogen dominance activity on the ectopic endometrial tissue.^33^ Progesterone resistance is due to the loss or alteration of receptor expression in the endometrium from women with endometriosis.^34^ Consequently, the reduction of 17-hydroxysteroid dehydrogenase type 2, normally induced by progesterone to convert E2 to the less potent estrone, and the upregulation of E2-producing p450 aromatase expression induce an E2 increased level in endometriosis tissue.^35^ Upregulation of E2 promotes the pain symptoms of endometriosis because of nerve growth and endothelial growth factors due to increased neuroangiogenesis and neuroinflammation.^36^

In light of the above, using a COC containing E2 could lead to fewer negative effects than EE on the endometriosis tissue.^37,38^ Unlike some other progestins, NOMAC is a 19-norprogesterone derivative that binds specifically to the progesterone receptor, exerts strong antiestrogenic effects, and has potent antigonadotropic activity; it reaches a peak serum concentration within 4 hours after oral administration, with a half-life of ∼50 hours.^39^ Because of its long half-life compared with other contraceptive progestins it is able to cover the 4-day hormone-free interval. Consequently, E2/NOMAC COC has a prevalent progestogen activity, reducing bleeding; moreover, amenorrhea increased somewhat over time with E2/NOMAC COC use.^40^

In our study 29.3% women had amenorrhea. Considering the retrograde flow of menstrual tissue through the fallopian tubes to be the most widely accepted explanation of endometriosis pathogenesis,^33^ the reduction or absence of bleeding in women on E2/NOMAC could be a useful effect.

Our study performed a clinical diagnosis of endometriosis-associated pelvic pain, but it is necessary to point out that to definitively diagnose the major forms of endometriosis, laparoscopy remains the gold standard. This could be a limitation of our study. However, today pelvic pain, when supposed to be due to endometriosis, can be treated without a definitive diagnosis using a hormonal drug to reduce menstrual flow, especially when women refer to be affected not only by chronic pelvic pain but also by dysmenorrhea and dyspareunia.^41^

It is important to consider that chronic pelvic pain usually starts during adolescence.^42^ In fact, most women affected by pelvic pain report symptoms beginning after menarche; they also report a longer time and worse experience before obtaining a diagnosis. Consequently, treatment is often started several years later, not always restricting the progression of endometriosis. Early diagnosis is essential to decrease pain and hopefully prevent disease progression and preserve future fertility.^43^ Usually the pain symptoms reappear when COC intake is discontinued. Consequently, its usage has to be for a long time. To choose a COC with natural estrogen, such as E2, and a progestogen with a strong receptor binding for ectopic endometrium, such as NOMAC, could avoid adverse metabolic events in users.

To enroll adolescents without a desire for pregnancy could be the aim of a future study to investigate the long-term effects of E2/NOMAC on pain and QoL, correlated to the metabolic aspects. This could permit us to better understand how to manage endometriosis-associated chronic pelvic pain.

## Conclusion

Women on E2/NOMAC COC reported a better reduction of endometriosis-associated chronic pelvic pain and an improvement of their QoL and sexual activity than those of women on NSAIDs. The current study has some limitations; the main one is the lack of laparoscopy to confirm endometriosis. Moreover, the lack of comparison between women on E2/NOMAC COC and women on COC having EE combined with a progestogen, rather than women on NSAIDs, or women on fixed dose of NSAIDs in a continuous regimen could be another limit of our investigation. Finally, a weakness of the study may be the lack of randomization and the fact that it was an unblended study. These limits need to be addressed in the future using a long-term follow-up investigation, to better analyze the end points of the current study.
